# Alignment theory of parallel-beam computed tomography image reconstruction for elastic-type objects using virtual focusing method

**DOI:** 10.1371/journal.pone.0198259

**Published:** 2018-06-15

**Authors:** Kyungtaek Jun, Dongwook Kim

**Affiliations:** 1 IM Technology Research Center, Seongbuk-gu, Seoul, Republic of Korea; 2 Department of Mathematics, Atlanta Metropolitan State College, Atlanta, Georgia, United States of America; Beijing University of Technology, CHINA

## Abstract

X-ray computed tomography has been studied in various fields. Considerable effort has been focused on reconstructing the projection image set from a rigid-type specimen. However, reconstruction of images projected from an object showing elastic motion has received minimal attention. In this paper, a mathematical solution to reconstructing the projection image set obtained from an object with specific elastic motions—periodically, regularly, and elliptically expanded or contracted specimens—is proposed. To reconstruct the projection image set from expanded or contracted specimens, methods are presented for detection of the sample’s motion modes, mathematical rescaling of pixel values, and conversion of the projection angle for a common layer.

## Introduction

X-ray computed tomography (CT) is an imaging technique in which the three-dimensional (3D) structure of a sample is reconstructed on the basis of two-dimensional projections formed by the penetration of X-rays at different projection angles. CT has become an essential technique in various fields, such as biology, archaeology, geoscience, and materials science [1–6].

In computer-based-imaging research, analysis of the elastic motion of objects has been largely restricted to the study of rigid-type-specimen motion. In such analysis, neither previous knowledge of the inherent differences, nor the image acquisition alone, are sufficient to determine the structures in the projection image set without some localized stretching or contraction of it. In such cases, the images are assumed to be obtained from specimens that simply must be rotated and translated with respect to one another to achieve the proper correspondence. However, it is difficult to reconstruct the projection image set from an object showing elastic motion on account of a certain constraint in continuity or smoothness that arises owing to the motion. The sample images from elastic objects may illustrate the same object as having different sizes depending on the projection angle during scanning. Consequently, mathematical modification is required to produce a consistently sized projection image set from the projection images of an object showing elastic motion. The modified projection image has an ideal sinogram pattern within each common layer. This issue has been addressed by employing an alignment solution, and various alignment solutions have been proposed [2, 7–12]. Furthermore, methods that improve the image quality that was previously degraded by amplified noise and artifacts have been developed [13–16]. Studies on the tracing of an object’s motion have also been conducted [17, 18].

Recently, Jun and Yoon proposed alignment solutions for CT image reconstruction using a fixed point and virtual rotation axis [7]. This approach provides a mathematical solution for the CT image reconstruction of a rigid-type object if the sample images have errors due to translation or tilting. The approaches using alignment solutions can be extended to address the reconstruction of the images projected from an object showing elastic motion.

In this paper, we provide mathematical solutions that can be applied to CT images to reconstruct the image of an object showing certain types of elastic motion, specifically periodic, regular, and elliptical expansion or contraction. Accordingly, we provide a technique to change the size of an object in a projection image. We focus on creating a rigid-type object by mathematically modifying a regularly or elliptically moving object in the case where the image cannot be obtained in a rigid-type form. Then, we reconstruct images by rescaling the pixel values in charge-coupled devices (CCDs) through mathematical correlations with the pixel value of the sample of the desired size (see Methods) and transforming them into an ideal sinogram pattern of a rigid-type specimen using an alignment solution with the virtual focusing method [7]. The main benefit of our method is that the sample size can be freely adjusted in the projection image set obtained from elastic-type objects showing the above motions. Image reconstruction is thus possible using alignments through a sinogram. We employ the reconstruction of the actual image sample as the experimental data and select a sample with a ring artifact to provide a mathematical solution.

## Sample motions and methods

### Basic concepts of the common layer

**Fig 1:**
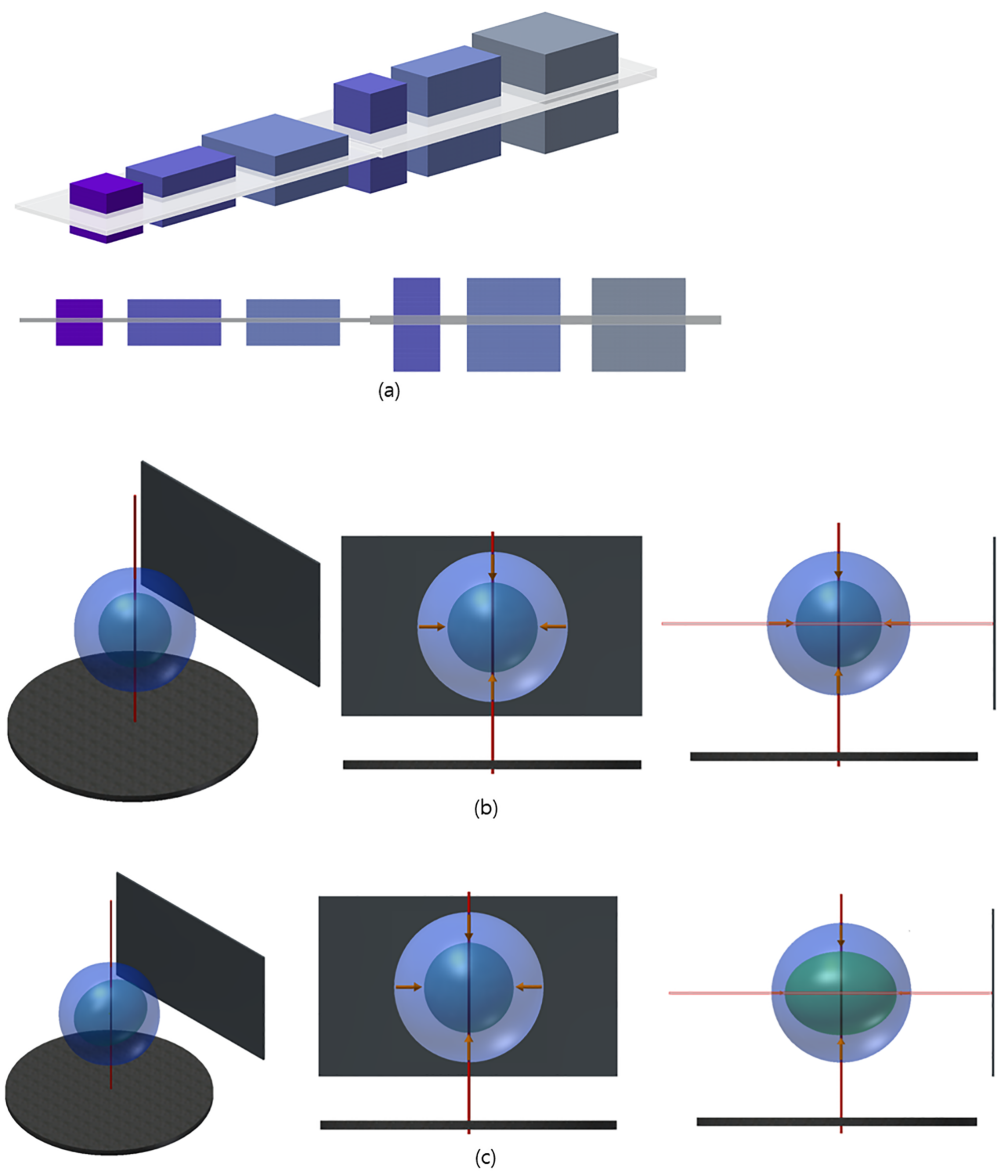
Change in the common layer for an elastic-type specimen and a schematic of two elastic specimens showing regular and elliptical contraction. (a) 3D image samples in space (left panel) and a front view. The gray part indicates the common layer. The first object (purple) is used to create five other objects by changing the size or height. The sizes of the first three samples are 10 × 10 × 10, 20 × 10 × 10, and 20 × 20 × 10, from the left, respectively. The next three samples are those whose heights from the first three samples have been doubled (10 × 10 × 20, 20 × 10 × 20, and 20 × 20 × 20, respectively). When the heights of the first three samples are doubled, the height of the common layer also doubles. (b) Schematic of a specimen that regularly contracts: 3D image (left), front view (middle), and side view (right). The common layer should be calculated for the thickness of the axial level considering the deformation rate of the object. (c) Schematic of a specimen that elliptically contracts. The figures are the 3D image (left), front view (middle), and side view with a middle axial level (right). The common layer should be calculated to be the same as (b) that with the elliptical deformation rate.

The common layer is a specific layer of an object that is common to all projection images in the object projection to obtain information. It is actually the axial level of the reconstructed space. The projection image for each angle must contain information about the same position of the same object. It is impossible to obtain the correct reconstruction image in the absence of the common layer. That is, a common layer is required to obtain a correct reconstruction image for an object. The projection image should contain a common layer, which must be secured before any reconstruction work is performed. The common layer for a rigid-type specimen was addressed in a previous study [7] (S1 Fig). For an elastic-type sample, the common layer that we herein address is shown in Fig 1a. In this study, we seek alignment solutions for an elastic-type specimen showing two motions: regular (Fig 1b) or elliptic contractions (Fig 1c) based on the common layer for elastic-type specimens.

The rigid-type specimen in a reconstruction only satisfies the Helgason–Ludwig consistency condition. The common layer should contain a common part of the height of the reconstructed object. Moreover, for a rigid-type specimen without a tilting error, it represents the same axial level of the projected specimen in the projection set. If the shape of the object changes during scanning, it may contain information about the same layer in some cases. However, if it changes in size in the axial direction, it should be considered to be a different object, and the information contained in it will also vary. Therefore, to reconstruct a projection image set of an object with a different size, it is necessary to convert it into an object with the same size. That is, the transformation of information should proceed in such a way that we have the same information as the object we are reconstructing. The projection set for the elastic specimen must be converted into the projection set for the rigid-type specimen.

It is a common idea that the voxels in the reconstructed space are related to specific parts of an object. To use a filtered inverse Radon transform that satisfies the consistency condition, it is necessary to determine the value related to the X-ray mass attenuation coefficient for the voxel in the projection image. This can be obtained by identifying the object part from which the voxel is transmitted. Since the reconstructed slice represents the cross-section of the specimen at a certain axial level, finding the corresponding portion of the voxels in the projection set is a necessary condition for creating an ideally focused reconstruction. In the case of an elastic-type object, it is necessary to align the projected specimens according to the unit length for the same axial level of the CCD. If the size of the object changes during scanning, it is necessary to adjust the projected specimen obtained at the same axial level (common layer) because a unit pixel in the CCD for each projection image may contain a different axial size in the specimen.

### Determination of sample motion modes

The deformation of elastic objects in mathematics can be modeled on the basis of three types of motion: motion in which the shape of an object changes periodically (periodic motion), motion in which the size is expanded or contracted while maintaining the shape (regular motion), or motion in which the size is expanded or contracted in a specific direction (elliptical motion). It is possible to apply the size changes in various directions through the study of the elastic object, which changes in size in a specific direction. Therefore, we here define motion modes in three categories.

In a sinogram without translation and tilt errors, all of the points of a rigid-type projected object satisfy the continuous sinusoidal function, *T*_*r*,*φ*_ (*θ*) = *r* cos (*θ* − *φ*) [7]. For an elastic-type object with regular motion, all points of the object move along sinusoidal functions *T*_*r*,*φ*_ (*θ*) = *r*(*θ*) cos (*θ* − *φ*) in the sinogram. Here, *r*(*θ*) is a contraction or expansion coefficient function. For an elastic-type object with elliptical motion, all of the points of the object move along sinusoidal functions *T*_*r*,*φ*_(*θ*) = *p*(*θ*) cos(*θ* − *φ*) cos(*α*) − *q*(*θ*) sin(*θ* − *φ*) sin(*α*), where *p*(*θ*) and *q*(*θ*) are the contraction or expansion coefficient functions of the major and minor axes, respectively, and *α* is the angle between the major axis of the elliptic specimen and the horizontal line of the CCD. Although accurate mathematical calculations are possible, the trajectory of a fixed point (high-density area) can be predicted and distinguished through the continuous sinusoidal function in the sinogram. Hence, we describe the motion modes using the trajectories of distinguishable high-density areas in the projection image.

**Fig 2:**
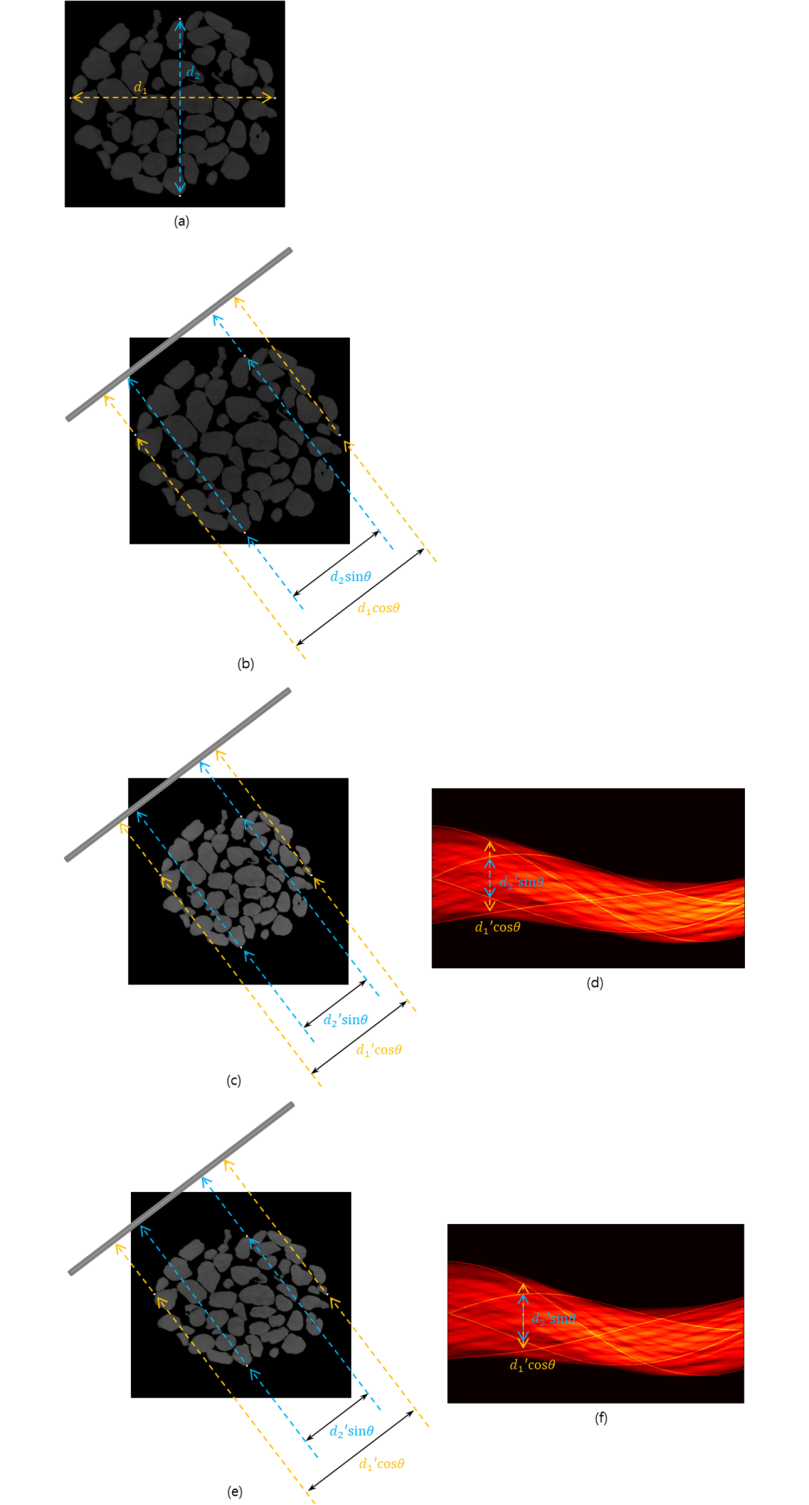
Determination of the motion modes using four fixed points. (a) Original image sample with four fixed points (high-density area). *d*_1_ is the distance between the left and right fixed points, and *d*_2_ is the distance between the top and bottom fixed points. (b) Distance between fixed points on the original image in the CCD. *θ* represents a projection angle. (c) Distance between fixed points on the regularly contracted image in the CCD. (d) Sinogram in the process of regular contraction. A high density in the sinogram represents the circular trajectory of the fixed points. (e) Distance between fixed points on the elliptically contracted image in the CCD. (f) Sinogram in the process of elliptic contraction. A high density in the sinogram represents the circular trajectory of the fixed points.

It is possible to determine the motion modes using four distinguishable high-density areas, which can be considered as fixed points (FPs). Fig 2 depicts the procedure for determining the motion types by measuring the distances among four FPs. Let us assume that there are four FPs, each of which is situated on the respective top, bottom, right, and left edges of a sample (Fig 2a). The distance between the right and left FPs is denoted by *d*_1_, and the distance between the top and bottom FPs is denoted by *d*_2_. For a projection angle *θ*, the two distances (*d*_1_ and *d*_2_) in the CCD can be represented by *d*_1_ cos*θ* and *d*_2_ sin*θ*, respectively (Fig 2b). Similarly, for the contracted image sample, these distances can be represented by *d*_1_′cos*θ* and *d*_2_′sin*θ*, respectively (Fig 2c and 2e). To determine the motion mode, it is necessary to measure the distance between FPs in the sinogram where the sample shows elastic motion. Once the desired size of the sample at a specific projection angle is determined, we can measure *d*_1_′cos*θ* and *d*_2_′sin*θ* from the sinogram (Fig 2d and 2f). The conditions for determining motion modes are as follows:

$$\frac{d_{1}\prime }{d_{1}}=k\frac{d_{2}\prime }{d_{2}}=\{\begin{array}{c} 1-\alpha (\theta ),\;when\;d_{1}>d_{1}^{\prime }\\ 1+\beta (\theta ),\;when\;d_{1}^{\prime }>d_{1} \end{array}$$ (1)

where *k* is the motion mode coefficient, *α*(*θ*) is the coefficient of contraction, and *β*(*θ*) is the coefficient of expansion. The elastic-type sample shows regular motion (contraction or expansion) if *k* = 1 and elliptical motion if *k* ≠ 1.

If the sample conserves its linearity, the projected center of attenuation (CA) can be used as an FP [7]. In this scenario, two FPs are sufficient to determine the mode of sample motion since *d*_1_ and *d*_2_ can be measured using the distances between the projected CA and the two FPs. It is also possible to use three FPs to numerically find the distance. However, when there are four FPs on the axis, the motion modes can be more accurately calculated.

### Specific motions

#### Periodic motion

In special cases, the change in an object shape may have periodicity, such as blood vessels that grow and contract in response to the heartbeat, and movements of the lung as it regularly expands and contracts. In these cases, the shape of an object that we reconstruct will appear periodic. If the periodicity is constant and the cycle can be recognized, a common layer can easily be obtained. For example, if the heart rate is constant at 120 beats per minute, we will obtain the same effect as taking a frozen heart or vein up to twice a projection every 0.5 s when we obtain information about the heart or its associated blood vessels. If the motion is fast for an X-ray dose, it is effective to take one image near a maximum or minimum with minimal change at every period of the periodic function. If the elastic specimen that we intend to reconstruct is in contact with other elastic specimens and is periodically affected by each adjacent specimen, we can obtain a projection of the rigid-type specimen in the same way in all common cycles of the specimens.

#### Objects that regularly expand or contract

First, we examine a case in which an object is regularly contracted (or expanded), but is not periodically contracted (or expanded) in the common layer during scanning. In this case, the shape of an object changes circularly or regularly at the same ratio. We can additionally consider the case in which the shape of an object is unknown because it is difficult to identify the periodicity. It is very difficult to predict that the size of an object will change during scanning because the shape will change depending on the projection angle at which the projection image is obtained. If there are two or more fixed points that contain a high-density area or a fiducial marker, they can be used to calculate the extent to which the specimen shape has changed [7, 19–24].

**Fig 3:**
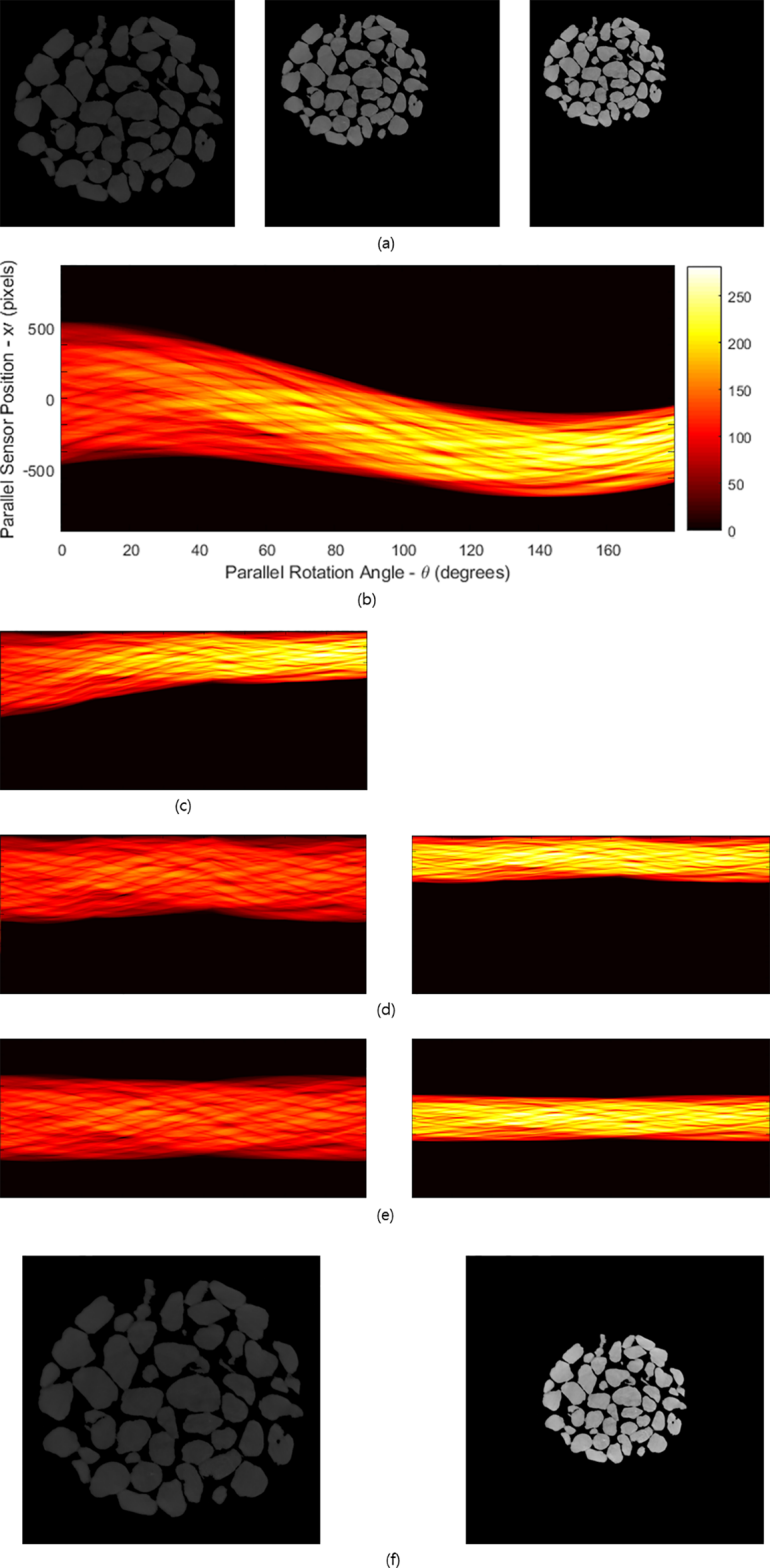
Reconstruction of an elastic-type object showing regular contraction that is continuous in time. (a) Process of deformation of an elastic-type object during scanning. Objects with different sizes are captured at different projection angles (0°, 90°, 180°). (b) Sinogram in the process of deformation. The amplitude of the sinogram gradually decreases with respect to the projection angle. (c) Sinogram modified by moving the projected shadow of the object to rescale the pixel values. (d) Sinogram after rescaling the pixel values to the contracted original-size sample (left) and to the final-size sample (right). (e) Convert non-ideal sinogram pattern into ideal sinogram pattern from each sinogram in (d) with a translation error using the virtual focusing method. (f) Ideally focused reconstructions from the sinograms in (e) for the original-size sample(left) and final-size sample(right).

Let us consider the case in which the size of an object regularly contracts with the same ratio. Fig 3a shows three samples with different sizes. They were captured at different projection angles (0°, 90°, 180°) during scanning. The object size is continuously reduced during scanning while maintaining the total mass attenuation coefficient (MAC) [7], which is measured using the unit voxel of a real specimen. The sinogram in the process of deformation for each projection angle is given in Fig 3b. The amplitude of the sinogram is gradually decreased with respect to the projection angle. In this simulation, we used 1,200 projected images with a contraction rate of 0.07% for each angle. That is, the projection angle changed 0.15° on the basis of the Nation Synchrotron Light Source X2B X-ray beamline at Brookhaven National Laboratory. However, discontinuity is apparent at each projection angle owing to the existence of a change in the density in two adjacent projection images. This implies that translation errors exist that must be resolved.

**Fig 4:**
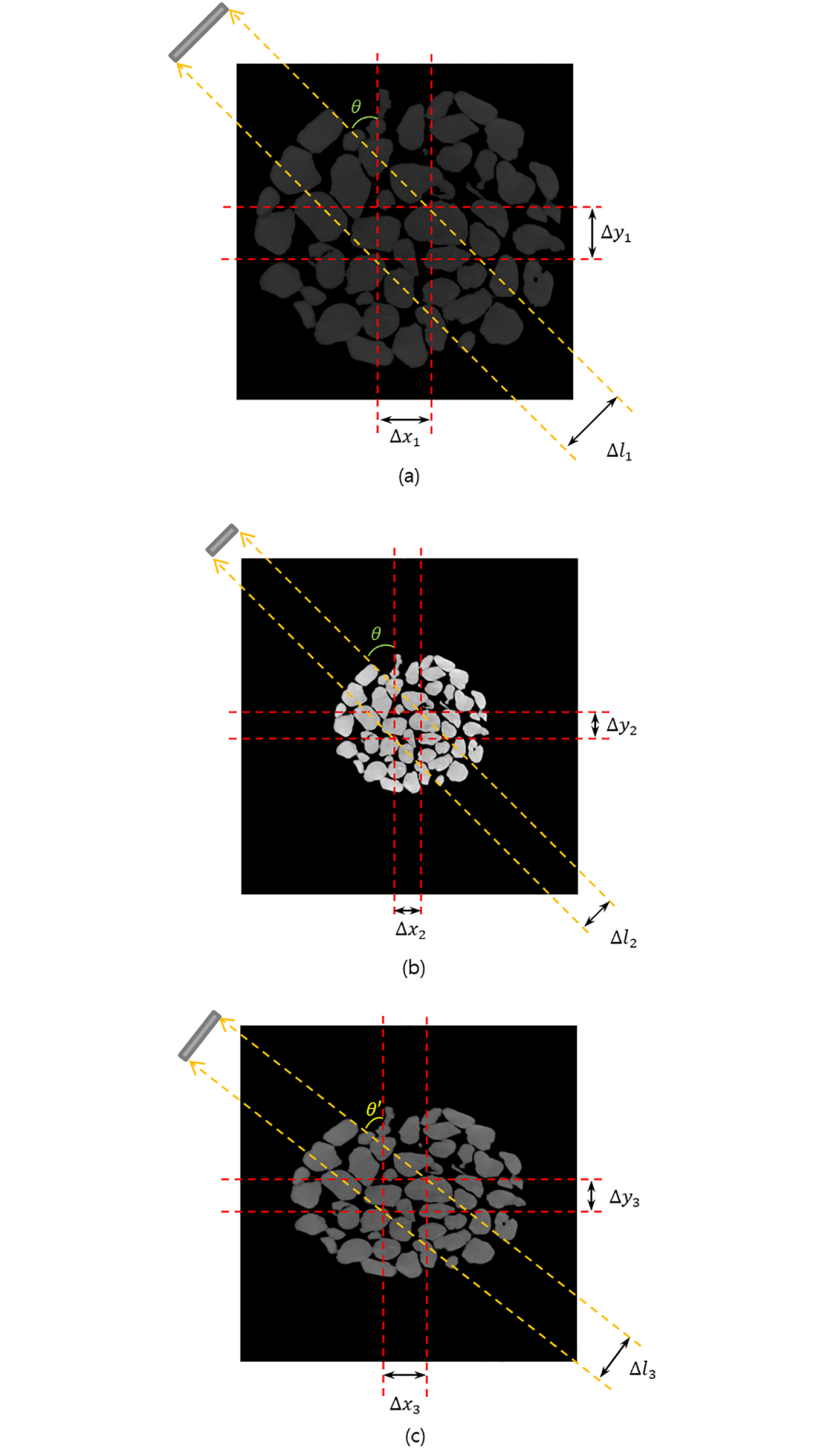
Changes in the CCD and the projection angle for the common area of the sample when X-rays penetrate samples of different sizes. (a) Original image specimen with a projection angle. When the X-ray for projection angle *θ* passes the crossed area of the image sample, the size of the area projected onto the CCD is Δ*l*_1_. (b) Regularly contracted image specimen. The X-ray projection angle for the common area does not change by *θ*; however, the size of the area projected onto the CCD decreases to Δ*l*_2_. (c) Elliptically contracted image specimen. The X-ray projection angle for the common area changes to *θ*′, and the size of the area projected onto the CCD is changed to Δ*l*_3_.

To reconstruct the image from the projection data, we should understand how the information is represented in each CCD in the process of obtaining the projection image. Fig 4a and 4b show the difference in the modified pixel value of a CCD for both the original image specimen (Δ*l*_1_) and contracted image specimen (Δ*l*_2_) for the common area. The CCD in the common area for the contracted image specimen receives larger pixel values of the CCD than the original. However, the information on the CCD of Δ*l*_1_ is the same as the information on the CCD of Δ*l*_2_. By understanding this relationship and reflecting it in the sinogram to adjust the size without changing the internal pattern, we can reconstruct the desired image (Fig 3, S2 and S3 Figs). Therefore, if we convert the linearly modified pixel values from the originally sized projection data into a pixel value in a column of a sinogram obtained from the desired size of the image specimen, we can obtain a sinogram obtained from the contracted image (Fig 3d, right).

Conversely, if we divide one set of linearly modified pixel values from the contracted image into linearly modified pixel values in a sinogram from the original image, we can obtain a sinogram for the size of the original image specimen (Fig 3d, left). After rescaling the pixel values, they must be corrected into ideal sinogram patterns by removing the translation errors using the virtual focusing method [7] (Fig 3e). Finally, the reconstructions will be obtained using the filtered inverse Radon transform (Fig 3f).

#### Objects that elliptically expand or contract

If the shape of an object changes elliptically rather than regularly, a more detailed method is needed to reconstruct its projection image set. Fig 4a and 4c show two image specimens. One is the original-size image specimen; the other is an image in which the object has elliptically changed in size.

In this case, it is also necessary to transform the original image specimen into an image with the desired size through modification of the sinogram. In the regular motion of an elastic-type sample, there is no change in the pattern of the sinogram according to the projection angle. However, if the size elliptically changes, the projection images at the same angle will not show the same pattern of the sinogram as the original one. To obtain the desired sinogram, it is first necessary to identify the correlation between the pattern for the projection angle obtained from the original image and that for the projection angle obtained from the deformed image. If a beam is projected from an object in the *θ* direction and the image is then elliptically contracted, the beam projected in the direction at a different angle *θ*′ passes through the same part of the object as the original image, and its pattern is also the same.

**Fig 5:**
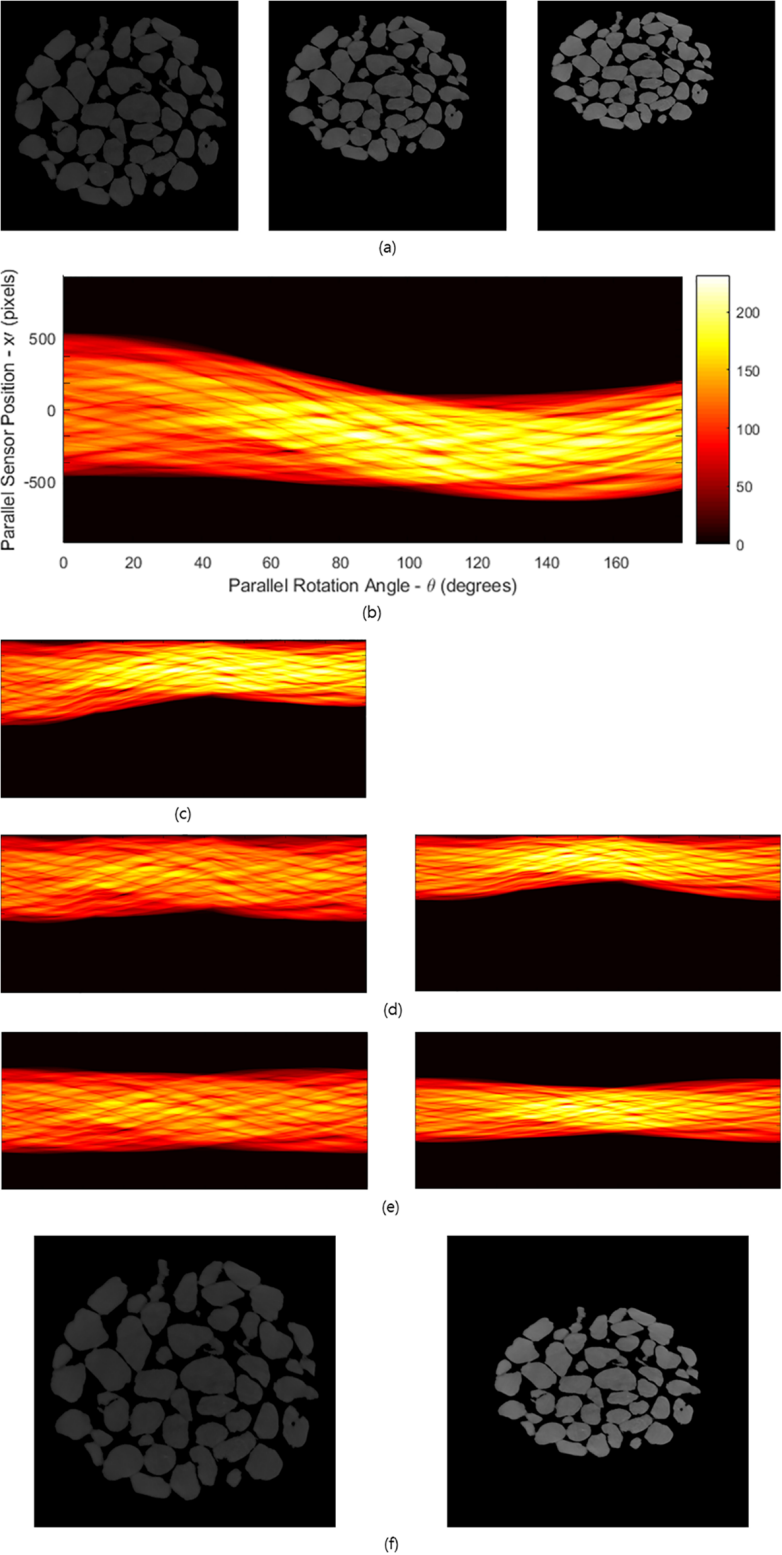
Reconstruction of an elastic-type object showing elliptical contraction that is continuous in time. (a) Process of deformation of an elastic-type object during scanning. Objects with different sizes are captured at different projection angles (0°, 90°, 180°). (b) Sinogram in the process of deformation. (c) Sinogram modified by moving the projected shadow of the object to rescale the pixel values. (d) Sinogram after rescaling the pixel values to the original-size sample (left) and to the contracted final-size sample (right). (e) Convert non-ideal sinogram pattern into ideal sinogram pattern from each sinogram in (d) with a translation error using the virtual focusing method. (f) Ideally focused reconstructions from the sinograms in (e) for the original-size samples (left) and final-size contraction (right).

Fig 5 shows the method for reconstructing projection images of objects showing elliptical motion. The captured objects at different angles (0°, 90°, 180°) are shown in Fig 5a. The sample contracts with the reduction rates of 0.05% in the *x* direction and 0.025% in the *y* direction. A sinogram for the elliptical motion of the sample is shown in Fig 5b. To reconstruct a projection image set, it is necessary to modify the projection image set for this angle using the method of *p* by *q* image sampling (see Methods). After modification, the sinogram obtained from the contracted object can be transformed into the sinogram of the original-size specimen (Fig 5d, left). It is possible to adjust the sinogram of a contracted object. Moreover, it is also possible to fit the sinogram produced by the original object to the contracted sinogram (Fig 5d, right). In other words, the size of the object can be adjusted to the desired size through modification of the sinogram. More generally, it is possible to resize the object in any direction. While obtaining the projection image of an object, we can reconstruct the image to the desired size if it is known the extent to which the object is stretched or shrunk horizontally and vertically.

### Virtual focusing method for elastic-type samples

**Fig 6:**
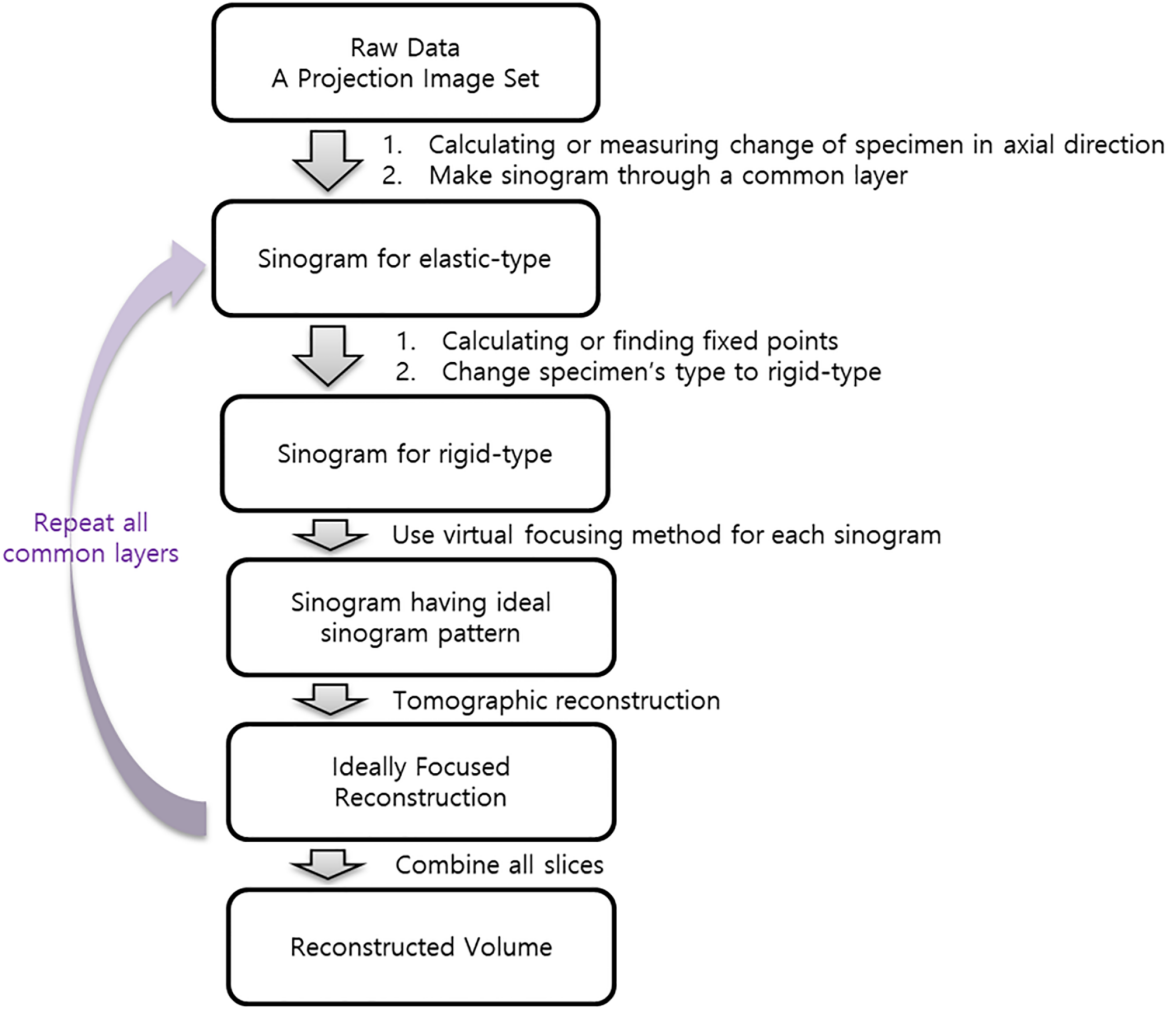
Flowchart of the process of obtaining a 3D reconstructed volume of elastic-type objects using the virtual focusing method.

By changing the projection image set of the elastic specimen to the projection image set of the rigid-type specimen, we can create an ideal sinogram pattern that satisfies the Helgason–Ludwig consistency condition. In this section, we present an approach to obtaining an ideally focused reconstruction of the two types of elastic models. A flowchart of this process is shown in Fig 6.

#### Virtual focusing method using a fixed point for rigid-type specimens

The circular trajectory of a point, *p*, in real space corresponds to a curve drawn by a sinusoidal function in the sinogram. The function is given by [7]

$$T_{r,\varphi }(\theta )=r\;\text{cos}\left(\theta -\varphi \right),\;0\leq \theta <180{}^{\circ}$$ (2)

where *r* is the distance between the rotation axis and point *p*, *θ* is the projection angle, and *φ* is the angle between line $\overset{⃡}{Op}$ and the line orthogonal to the X-ray direction (S4 Fig).

In ideal cases, the center, *O*, is converted into *T*_0,*φ*_ in the sinogram but not in the actual sinogram. *T*_*r*,*φ*_ is a function that shows how a specific point *p* in real space moves on the sinogram. Hence, if point *p* in the solid specimen rotates on the stage, and the projected curve drawn by the movement of *p* for each angle is the same as the sinusoidal curve by *T*_*r*,*φ*_ in the sinogram, then the projected trajectories of the other points in the specimen should satisfy the projected curves by $T_{r_{n},\varphi_{n}}$.

#### Regular motion algorithm (*p* by *p* contraction)

A specimen showing regular elastic motion has no change in its internal pattern, even if it changes in size. In this case, we can change the size of the original projection image to the size of the contracted projection image by rescaling the modified pixel values of the CCD. Furthermore, we can orient the contracted projection image size in the opposite direction to the original projection image size. Therefore, to achieve ideally focused reconstruction, the following process is followed:

**Step 1**: In the regularly contracted image set, we change the projection set obtained at the original density to the projection set represented by the MAC.
**Step 2**: Since the original projection image is less dense than the contracted projection image, the MACs of the projection image (the column of the sinogram) should be rescaled to the desired reconstruction size for the desired projection angle in the projection image set (S2 Fig).
**Step 3:** Using the fixed point and the virtual focusing method, the non-ideal sinogram pattern obtained from specimens of different sizes is transformed into the ideal sinogram pattern.
**Step 4:** An ideally focused reconstruction can be obtained from the sinogram.

#### Elliptical motion algorithm (*p* by *q* contraction)

In the case of an elliptical elastic specimen, even if the projection angle is the same, different patterns can be observed among the projection images when the size of the specimen is different. Between the elliptically contracted projection image and the original projection image, there is a different pattern at a specific angle. However, mathematical modification at a particular projection angle can be used to make the pattern of the elliptically contracted projection image the same as that of the original projection image. From that point, the same projection image can be made by rescaling the modified pixel values of the CCD. Therefore, to achieve ideally focused reconstruction, the following process is followed:

**Step 1**: In the elliptically contracted image set, we change the projection set obtained at the original density to the projection set represented by the MAC.
**Step 2**: We convert the MACs of the projection image to the desired reconstruction size for the desired projection angle and obtain a new projection angle through the projection angle before transformation. To obtain a new projection angle in the case of a specimen with a *p* × *q* contraction from the initial specimen size, the following equations are needed. If the projection angle before the change passes through (1, *m*) from the origin, then the transformed projection angle passes through (*p*, *qm*). Accordingly, we can know a new projection angle $\theta^{\prime }={tan}^{-1}(\frac{qm}{p})$. Moreover, the way in which the size is transformed between projection images using two angles can be calculated using the following equation:
  $$l_{1}=\frac{\left|m+1\right|}{\sqrt{m^{2}+1}},\;l_{2}=\frac{\left|pm^{\prime }+q\right|}{\sqrt{({m^{\prime })}^{2}+1}}$$ (3)
  where $m^{\prime }=\frac{qm}{p}$, *l*_1_ and *l*_2_ are the distance from a line with the same slope as the projection angle through the origin to (1, −1), and the distance from a line with the same slope as the projection angle through the origin to (*p*, −*q*) on the CCDs (Fig 4c), respectively. We rescale the modified pixel values of a CCD using the ratio of Δ*l*_1_ and Δ*l*_2_.
**Step 3**: Using the fixed point and the virtual focusing method, the non-ideal sinogram pattern obtained from the elliptically expanded projection image is transformed into the ideal sinogram pattern.
**Step 4**: An ideally focused reconstruction can be obtained from the sinogram.

## Results and discussion

Through the sinogram adjustment, an expanded or contracted reconstruction image of an elastic-type object can be obtained. Objects that regularly change in size require modification of the length in the sinogram, while objects that elliptically change require transformation of the unit pixel length and the projection angle in the sinogram. When modifying the projection angle, the spacing of the projection angles may not be equal. This does not cause a significant problem because inverse Radon transformation is defined for projection angles of unequal spacing. However, if the image is considerably expanded in one direction and a projection set is obtained for a change in equally spaced projection angles, the projection angle becomes dense at a certain angle. In this case, the image may be slightly blurred by the filter function. Further research on the filter function is needed (S5 Fig).

The reconstruction of the expanded object appears darker than the original image (owing to the lower MAC). However, if the projection image has linearity in the absorption of photons and in the specimen thickness, a clearer image can be obtained by adjusting the constant of MAC. The resulting image has no changes in the pattern before the adjustment or in the shape of the image. This result suggests that, when we irradiate the object, we can reduce the radiation dose within the digitally adjustable limits [7]; that is, there is no reason to increase the radiation dose of the object more than necessary, and the radiation image can be obtained by using a lower amount of radiation. This result is very significant, as a lowered amount of radiation can reduce the damage in cases where the radiation dose is harmful, especially in the case of living tissue.

We used image samples with ring artifacts to check the incidence of errors in our method. In an image with a ring artifact, the pattern in the image specimen remains unchanged. It is difficult to precisely observe the ring artifact if the rotation axis is misaligned or not ideally focused in the reconstruction. In the case of regular expansion, contraction, and periodic motion, reconstruction can be performed by adjusting the size to the desired dimensions, even if the projection images for various specimens of different sizes are mixed.

**Fig 7:**
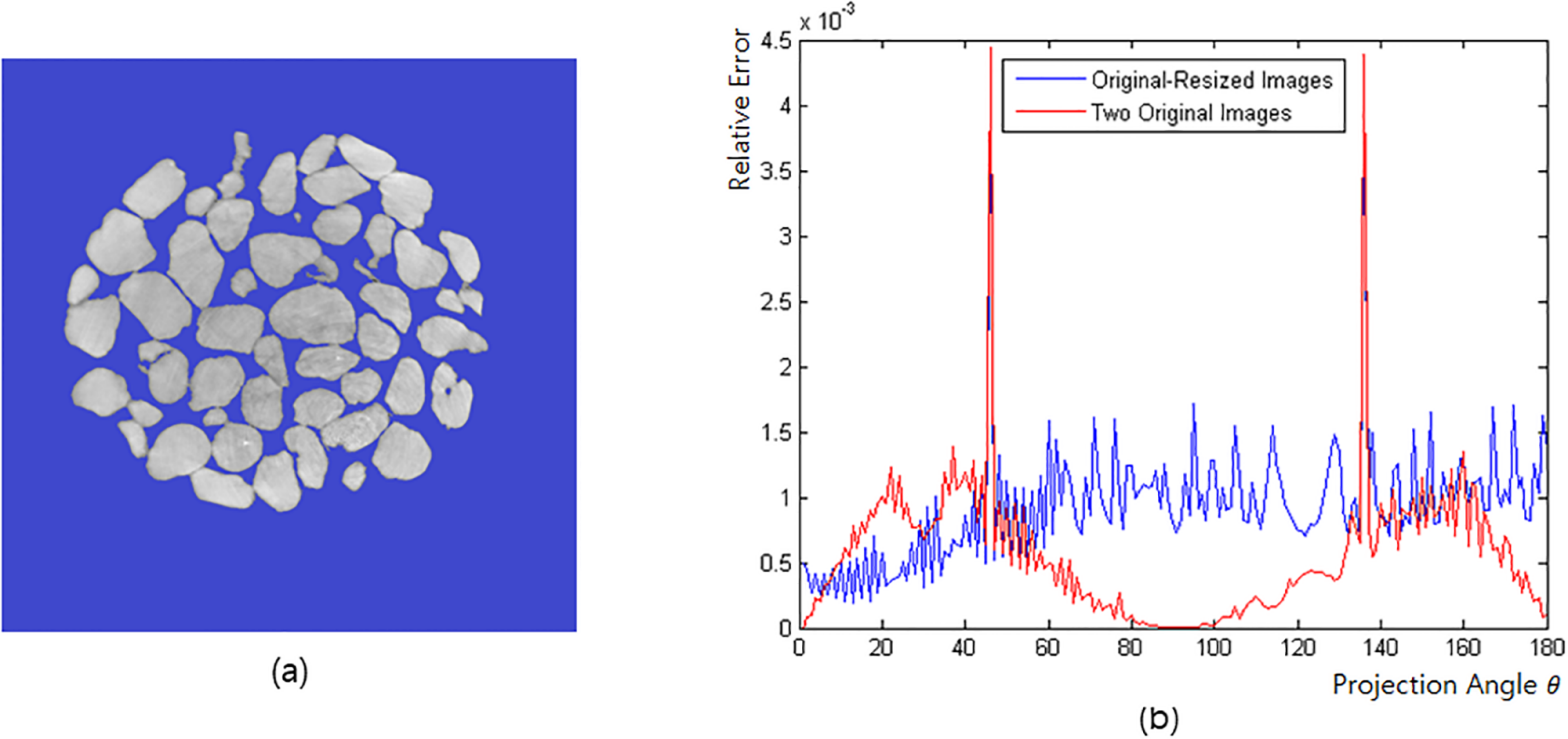
Difference in the relative errors. (a) 746 × 746 image sample, where the gray part is the region of interest. (b) Blue curve is the relative error that is calculated after the CA of the resized projection image is transformed into the CA of the original projection image. The red curve is the relative error that is calculated when the CA of the original-size projection image moves one voxel and the CA of the original-size projection image does not move.

In the case of elliptic expansion, contraction, and periodic motion, it is possible to obtain an ideally focused reconstruction by rescaling the modified pixel values of the CCD and the projection angles of each projection image for specimens with different sizes (Fig 5). Using an ideal sinogram and a resized sinogram, we can create a contracted image that is within the error range (Fig 7b). Therefore, the reconstruction created by resizing the projection image results in an image without errors.

It is not easy to obtain the ideally focused reconstruction of living cells or organs because of their movements. It requires significant effort to obtain a better reconstruction during scanning because the ideally focused reconstruction only occurs when the cells are fixed or have a specific shape. However, using the method presented herein, it is expected that an ideally focused reconstruction will be obtained without lethal damage or manipulation of living cells or organs.

It has been proposed that the modified pixel value of the projection image should be changed to a value that is linearly proportional to the length of the specimen associated with the MAC. This implies that the CA plays the role of an FP and has a notable effect on the pixel value of the reconstruction. If we use a projection image set that does not have linearity, we will have a different reconstruction from the original specimen, and mathematical correction will not be possible (see S6 Fig). When projecting images of human body parts, such as the heart and lungs, they are affected by high-density areas such as bones at certain projection angles. This is a concern because it can result in images that are different from the actual image. Our technology requires more research before it can be applied to the processing of X-ray images of the human body.

We additionally investigate the difference in the relative errors that are calculated (1) after the CA of the resized projection image is transformed into the CA of the original projection image and (2) in the scenario in which the CA of the original-size projection image moves one voxel and the CA of the original-size projection image does not move. The relative error can be calculated using the following equation:

$$E(\theta )=\frac{\sum_{k}\left||{P1}_{\theta }(k)-\;{P2}_{\theta }(k)|\right|}{N}$$ (4)

where *N* is the total number of pixels in the region of interest, and *P*1_*θ*_ and *P*2_*θ*_ are the original projection image and changed projection image, respectively.

Fig 6 shows the sample image, where the gray part is the region of interest (left), and the two relative errors occur in terms of the projection angle (*θ*; right). In the right panel, the blue curve is the relative error of (1); the red curve is the relative error of (2). A difference exists from the specific projection angle depending on the image. In the projection image, a peak occurs when a high-density pixel is severely divided by the angle (S7 Fig). In general, the relative error has the property that a smaller relative error is obtained when the image is larger. The relative error of the resized density is similar to the maximum value of the relative error, which is different from the actual value of one pixel; the relative error continues on the basis of the position. If the pixel size is reduced, the relative error will also be reduced.

## Supporting information

S1 Fig Correlation between the common layer and axial level. The common layer is generally the axial level of the reconstructed 3D object with the mass attenuation coefficient or density. (a) Common layer for a rigid-type specimen moves up when the object has a translation error in the direction of the axial level. The common layer also moves as the specimen moves. (b) If the rotation axis is vertically tilted, the common layer rotates along the trajectory of each part of the specimen rotated by the stage.
(TIF)

S2 FigRescaling image with linear interpolation: Resizing pixels 3 and 4 with the same total attenuation. In this case, the third value of the expanded pixel value P2_*θ*_(3) is calculated by ${P1}_{\theta }(2)*\frac{2}{4}+{P1}_{\theta }(3)*\frac{1}{4}$.(a) Original projection image *P*1_*θ*_ with the pixel values. (b) Expanded projection image *P*2_*θ*_ with the rescaled pixel values.(TIF)

S3 Fig Basic description for the expanded voxel from the original voxel. (a) Example of regularly doubled expanded voxels. Each bolded arrow indicates X-ray penetration in sections *s*_1_ and *s*_2_, and their region will be doubled. (b) Basic information table.
(TIF)

S4 FigSinograms of circular image specimen and its reconstructions. The stage is marked with a red dot at the bottom to indicate θ = 0°.(a) Sinogram when the specimen (right panel) is translated in parallel with the beam from the center of the stage at θ = 0°. (b) Sinogram and its reconstruction with which we determined $\overset{⃑}{P_{CA}}$ of each column in the sinogram in (a) and aligned them on function T_50,30°_. $\overset{⃑}{P_{CA}}$ is marked in black on each column of the sinogram. Because $\overset{⃑}{CA}$ is off the center of the stage, the black line shows a sinusoidal function. The reconstruction is moved to the upward side of the original stage. (c) Sinogram and its reconstruction with which we determined $\overset{⃑}{P_{CA}}$ of each column in the sinogram in (a) and aligned them on function T_0, φ_. $\overset{⃑}{P_{CA}}$ is marked in blue on each column of the sinogram. Because $\overset{⃑}{CA}$ is moved to the center of the stage this time, it appears as a straight line across the center. The center of the specimen reconstruction is moved to the center of the original stage. This modification shows the same result as moving the specimen in real space.(TIF)

S5 FigElliptically expanded reconstruction from the original projection image set. If the ideal sinogram with a uniformly changed projection angle is modified to a sinogram of an image that is severely expanded in the *x* direction, the ideally focused reconstruction is not clear.(a) Specimen. (b) Ideal sinogram of (a). (c) Sinogram that is elliptically doubled in the *x* direction from the sinogram in (b). (d) Translation of each $\overset{⃑}{P_{CA}}$ in the columns of the sinogram onto *T*_0, *φ*_. (e) Ideally focused reconstruction obtained from (d).(TIF)

S6 Fig Change in the reconstruction according to the relationship between the X-ray path length and the density for a circle with a radius of 100. (a) Three types of density functions related to the X-ray path length. The red line shows a linear relationship. (b) Sinogram and its reconstruction using the linear relationship from the red line in (a). (c) Sinogram and its reconstruction using the relationship from the blue curve in (a). (d) Sinogram and its reconstruction using the relationship from the green curve in (a).
(TIF)

S7 Fig Example when the worst-case relative error occurs because of the limitations of the digital image. (a) Specimen on a stage at two different positions. The specimen is placed at the center of the stage (left panel). The specimen moves from the center to the right by 0.5 voxel (right panel). (b) Translation of the right projection image, which has the same projected CA position of the first projection image in (a). In this case, the relative error is one.
(TIF)
