# Supplementary figures and images for: Alignment theory of parallel-beam computed tomography image reconstruction for elastic-type objects using virtual focusing method
Source: PLoS One. 2018 Jun 15;13(6):e0198259. doi: 10.1371/journal.pone.0198259 (PMC6003809; doi:10.1371/journal.pone.0198259)

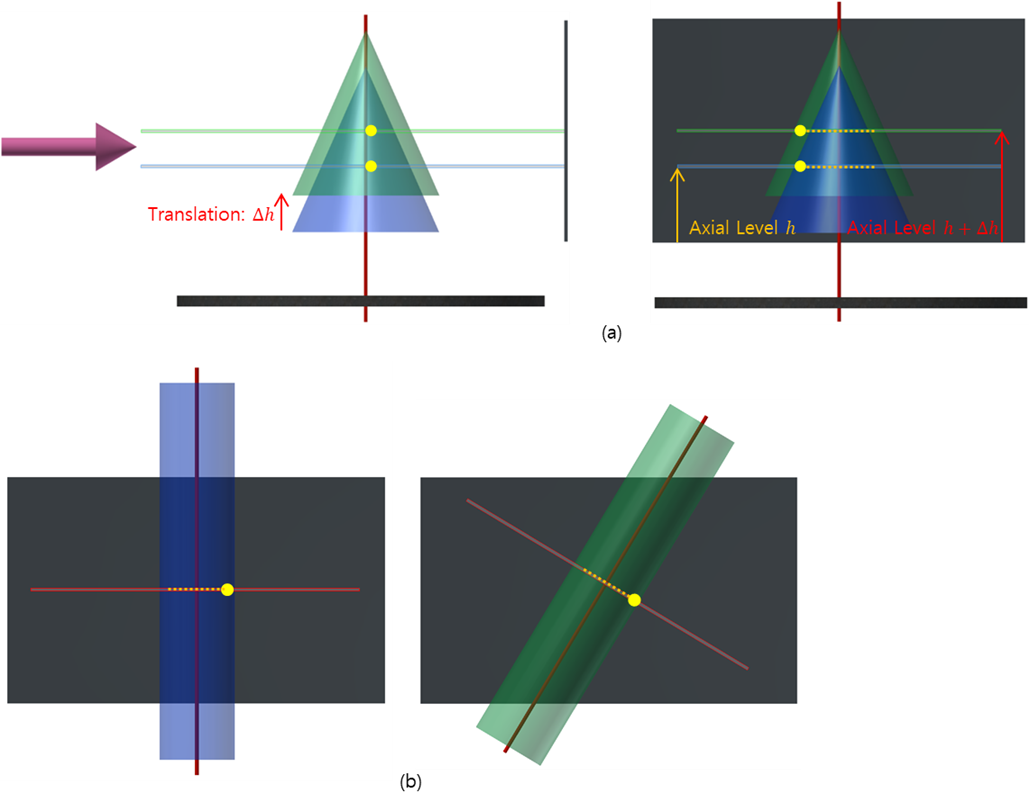

Supplement: S1 Fig — (a) Common layer for a rigid-type specimen moves up when the object has a translation error in the direction of the axial level. The common layer also moves as the specimen moves. (b) If the rotation axis is vertically tilted, the common layer rotates along the trajectory of each part of the specimen rotated by the stage. (TIF) [file pone.0198259.s001.tif]

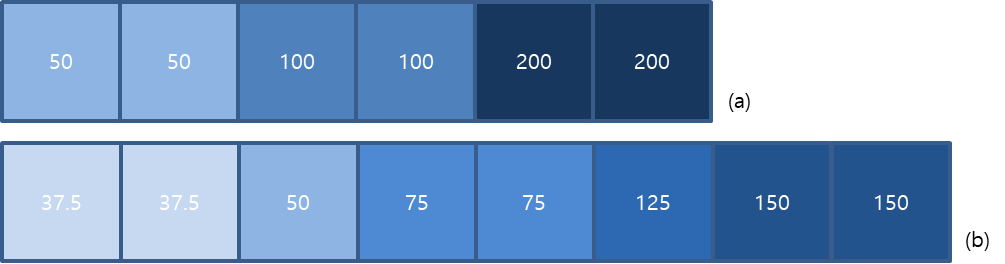

Supplement: S2 Fig — (a) Original projection image P1θ with the pixel values. (b) Expanded projection image P2θ with the rescaled pixel values. (TIF) [file pone.0198259.s002.tif]

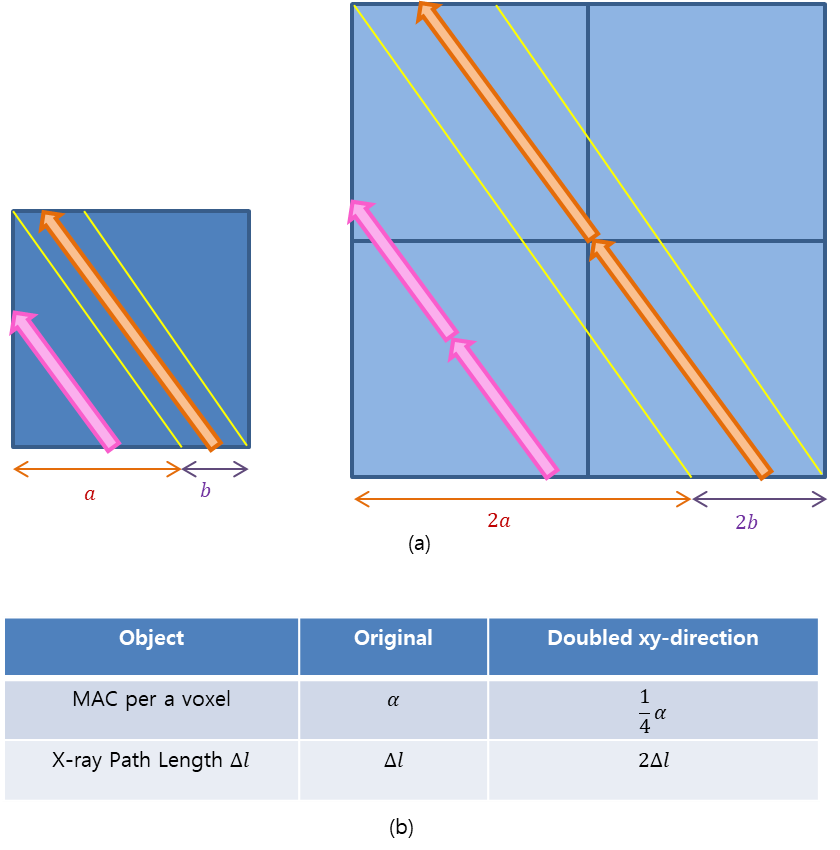

Supplement: S3 Fig — (a) Example of regularly doubled expanded voxels. Each bolded arrow indicates X-ray penetration in sections s1 and s2, and their region will be doubled. (b) Basic information table. (TIF) [file pone.0198259.s003.tif]

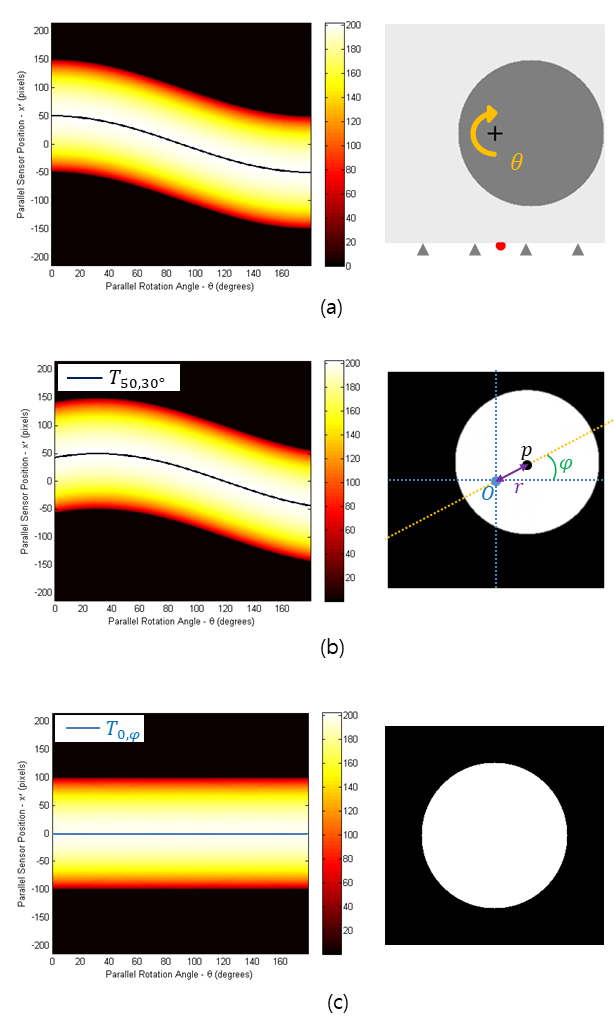

Supplement: S4 Fig — (a) Sinogram when the specimen (right panel) is translated in parallel with the beam from the center of the stage at θ = 0°. (b) Sinogram and its reconstruction with which we determined PCA⃑ of each column in the sinogram in (a) and aligned them on function T50,30°. PCA⃑ is marked in black on each column of the sinogram. Because CA⃑ is off the center of the stage, the black line shows a sinusoidal function. The reconstruction is moved to the upward side of the original stage. (c) Sinogram and its reconstruction with which we determined PCA⃑ of each column in the sinogram in (a) and aligned them on function T0, φ. PCA⃑ is marked in blue on each column of the sinogram. Because CA⃑ is moved to the center of the stage this time, it appears as a straight line across the center. The center of the specimen reconstruction is moved to the center of the original stage. This modification shows the same result as moving the specimen in real space. (TIF) [file pone.0198259.s004.tif]

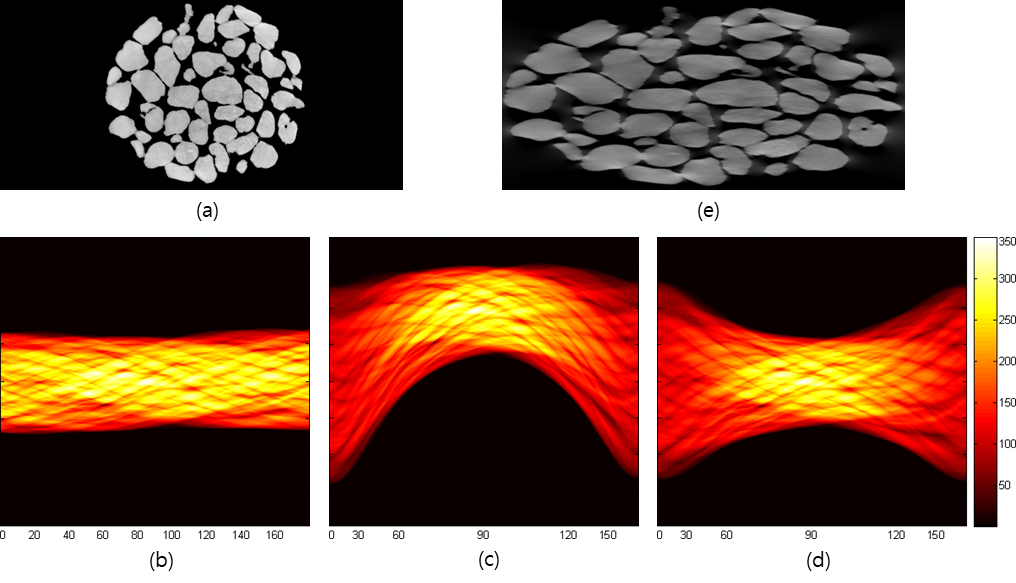

Supplement: S5 Fig — (a) Specimen. (b) Ideal sinogram of (a). (c) Sinogram that is elliptically doubled in the x direction from the sinogram in (b). (d) Translation of each PCA⃑ in the columns of the sinogram onto T0, φ. (e) Ideally focused reconstruction obtained from (d). (TIF) [file pone.0198259.s005.tif]

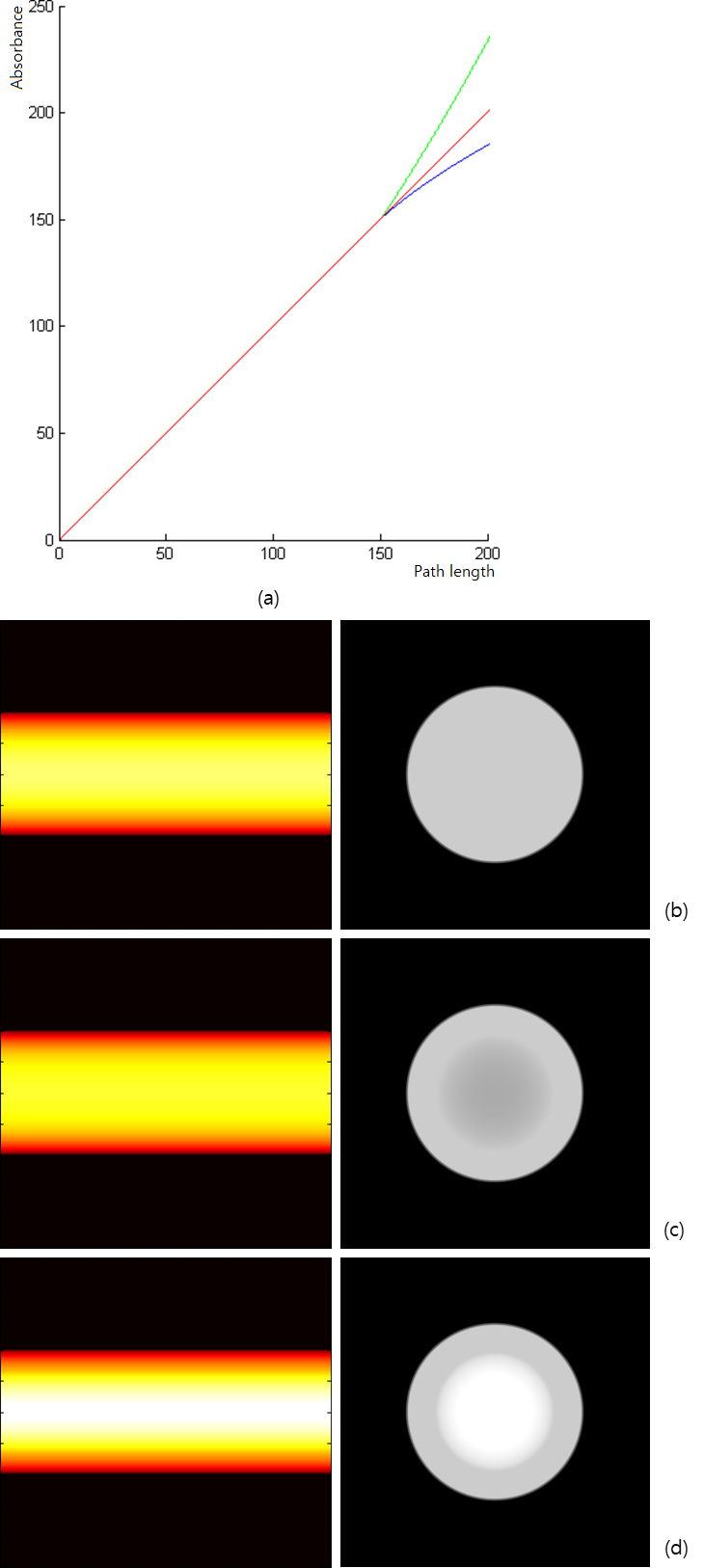

Supplement: S6 Fig — (a) Three types of density functions related to the X-ray path length. The red line shows a linear relationship. (b) Sinogram and its reconstruction using the linear relationship from the red line in (a). (c) Sinogram and its reconstruction using the relationship from the blue curve in (a). (d) Sinogram and its reconstruction using the relationship from the green curve in (a). (TIF) [file pone.0198259.s006.tif]

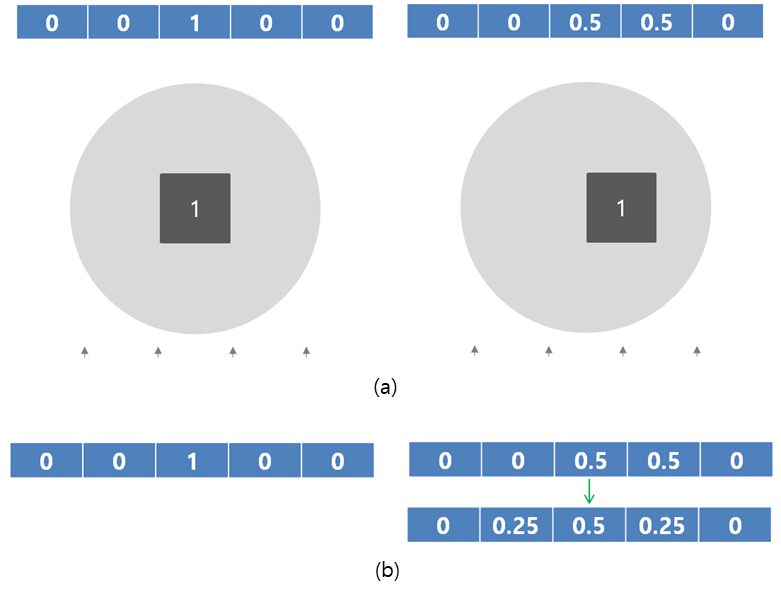

Supplement: S7 Fig — (a) Specimen on a stage at two different positions. The specimen is placed at the center of the stage (left panel). The specimen moves from the center to the right by 0.5 voxel (right panel). (b) Translation of the right projection image, which has the same projected CA position of the first projection image in (a). In this case, the relative error is one. (TIF) [file pone.0198259.s007.tif]
